# The Practice Market
*The previous articles in this series appeared in The Hospital of March 18, 25. and April 1.


**Published:** 1911-04-29

**Authors:** 


### SPECIAL ARTICLES.

### THE PRACTICE MARKET.*

### IV. — MEDICAL PARTNERSHIP AGREEMENTS.

In our last article we considered the taking in of a partner from the vendor's point of view. In most cases the ideal partner is a youngish man—well up in his work and well qualified, of good appearance and manner, with some experience of private practice, and personally known beforehand to the vendor or his friends. Candidates who satisfy all these conditions except the last are not infrequently to be found upon the books of first-class agencies, but, as a rule, the best partners are obtained privately.

When a new partner is introduced into a medical firm, however closely the contracting parties may be related by blood or friendship, a full formal partnership agreement is absolutely necessary. Any solicitor who knows his work ought to be able to draw up a satisfactory deed of partnership between two or more medical practitioners. But in medical partnership agreements there are technical points, peculiar to the practice of medicine in its business aspects, or arising out of the traditions and ethics of the profession, which are everyday matters to a medical agency or a firm of London solicitors accus-

* The previous articles in this series appeared in The Hospital of March 18, 25, and April 1.

Page(s) missing

